# Potassium channels in cell cycle and cell proliferation

**DOI:** 10.1098/rstb.2013.0094

**Published:** 2014-03-19

**Authors:** Diana Urrego, Adam P. Tomczak, Farrah Zahed, Walter Stühmer, Luis A. Pardo

**Affiliations:** 1 Oncophysiology Group, Max Planck Institute of Experimental Medicine, Hermann-Rein-Strasse 3, Göttingen 37075, Germany; 2 Department of Molecular Biology of Neuronal Signals, Max Planck Institute of Experimental Medicine, Hermann-Rein-Strasse 3, Göttingen 37075, Germany; 3 Cluster of Excellence Nanoscale Microscopy and Molecular Physiology of the Brain, Göttingen, Germany

**Keywords:** potassium channels, proliferation, cell cycle, cell division, cancer

## Abstract

Normal cell-cycle progression is a crucial task for every multicellular organism, as it determines body size and shape, tissue renewal and senescence, and is also crucial for reproduction. On the other hand, dysregulation of the cell-cycle progression leading to uncontrolled cell proliferation is the hallmark of cancer. Therefore, it is not surprising that it is a tightly regulated process, with multifaceted and very complex control mechanisms. It is now well established that one of those mechanisms relies on ion channels, and in many cases specifically on potassium channels. Here, we summarize the possible mechanisms underlying the importance of potassium channels in cell-cycle control and briefly review some of the identified channels that illustrate the multiple ways in which this group of proteins can influence cell proliferation and modulate cell-cycle progression.

## 1. Introduction

Regulation of cell division is of great relevance for eukaryotes. Cells must proliferate throughout ontogenesis, tissue renewal and remodelling, and to repair damaged areas during wound healing. Defective cell-cycle checkpoints are a common feature of cancer cells and the inactivation of cell cycle regulators decides the physiological or pathological fate of stem cells. Although there are a large number of studies on the molecular and biochemical mechanisms controlling the cell cycle, the bioelectrical modulation of cell-cycle progression is still poorly understood. K^+^ channels have been implicated in the control of cell-cycle progression both through their influence on the membrane potential and non-canonical, permeation-independent mechanisms.

## 2. Checkpoints and transmembrane potential regulate cell-cycle progression

The process that produces two daughter cells from a mother cell has been divided into several phases, each with very characteristic functional properties. Cell division in eukaryotes starts with the G1 (*gap* 1) phase, which separates the previous cell division from the period of DNA synthesis (S-phase), where chromosome replication is accomplished. This is followed by the second gap (G2) and the mitotic (M) phase. After M phase, a cell can proceed to a new G1 phase or enter a quiescent state (termed G0) that can last for a very long time, even for the rest of the life of the cell in the case of end-differentiated cells. The correct progression of the cycle is guaranteed because the initiation of a late event is strictly dependent on the successful completion of the preceding step. In eukaryotic cells, for example, mitosis will not start until the completion of DNA synthesis. The interdependency of events is owing to a series of surveillance or control mechanisms termed checkpoints, which have evolved to minimize the production and propagation of genetic inaccuracies [1,2]. The complex machinery of cell-cycle checkpoints includes in all cases a sensor supervising the completeness of a particular task and a response element triggering the next downstream event, which will be a process involved in the actual replication and segregation of the DNA. For instance, the downstream event at the onset of S phase is DNA synthesis, the downstream event at the onset of mitosis is the assembly of the spindle and the downstream event at the end of mitosis is chromosome segregation [3,4]. Thus, checkpoints are constitutive feedback control pathways safeguarding key cell-cycle transitions G1/S, G2/M and exit from mitosis [5]. The key components of the mechanisms coordinating the downstream events are cyclin/cyclin-dependent kinase (CDK) complexes, which need to be expressed in a timely fashion and/or activated to allow cell-cycle progression.

**Figure 1.**
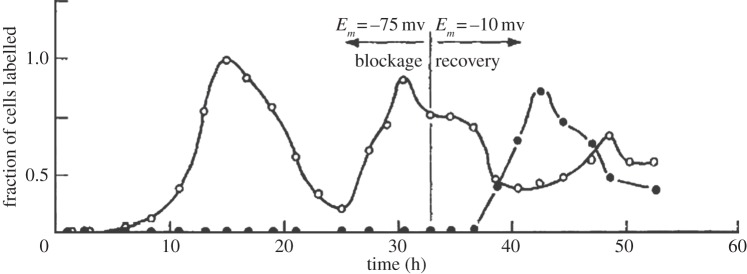
Complete block of DNA synthesis, measures as [^3^H]thymidine incorporation in cells with fixed hyperpolarized membrane potential. Reproduced from [7] with permission. Open circles, control; black circles, manipulation of membrane potential.

The transmembrane potential has been reported as a cellular bioelectric parameter that influences the progression through the cell cycle [6]. The concept came from the early experimental observation of a correlation between the resting membrane potential and the degree of mitotic activity [7]; forcing the membrane potential of Chinese hamster ovary cells to a fixed hyperpolarized value completely inhibited DNA synthesis measured as [^3^H]thymidine incorporation, while cycling was recovered upon release of the potential (figure 1). Cell types with a very hyperpolarized resting potential, such as muscle cells and neurons, typically show little or no mitotic activity. Inversely, it was reported in the early 1970s that ouabain-induced depolarization was followed by the initiation of DNA synthesis and subsequent mitosis in chick spinal cord neurons [8,9]. Moreover, it has been shown that the membrane potential is not constant during progression through the cell cycle [10,11]. For example, the distribution of membrane potentials in cells from the breast cancer cell line MCF-7 is multimodal. The frequency of events at each maximum can be shifted when experimental treatments change the distribution of cells among the different phases of the cell cycle. The results of these experiments showed a pattern of positive correlation where the membrane potential hyperpolarizes during the G1/S transition, there is a significant contribution of depolarized cells towards G0/G1 and an enrichment in hyperpolarized cells towards G2/M transition [12].

## 3. K^+^ channels as important players in the cell cycle

**Figure 2.**
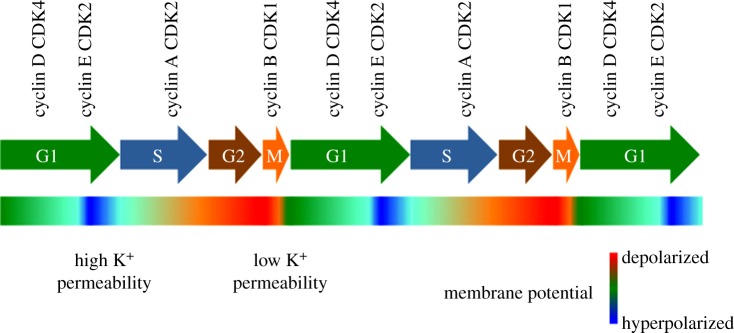
Schematic of the behaviour of the membrane potential along the cell cycle. Different potassium channels show variations of expression or activity through the cell cycle, thus shifting the membrane potential towards hyperpolarized values, close to the equilibrium potential for potassium, at the border between G1 and S-phases.

If the membrane potential is not constant along the cell cycle, cell-cycle-dependent changes in membrane permeability are required (figure 2). Potassium conductance governs the resting membrane potential in both excitable and non-excitable cells. In contrast to an action potential fired by a neuron, the potential changes along the cell cycle are much slower, gradual and smaller, and can be intuitively explained by modifications in the conductance that sets the resting membrane potential. Proliferation was one of the first identified aspects of cell physiology where potassium channels play a crucial role. The early observation that wide-spectrum potassium channel blockers inhibit proliferation [13] has been repeatedly confirmed in many tissues and cell types (reviewed e.g. in [6]). Many different potassium channels show cell-cycle-dependent variations of expression or activity [14–17].

For instance, a large conductance, voltage-gated K^+^ channel is expressed in unfertilized mouse oocytes; in the first cell cycle of fertilized oocytes, the channel is active throughout M and G1 phases, and inactive during S and G2. Thus, changes in channel activity set the membrane potential along the cell cycle in the oocyte [18]. Increasing evidence shows that voltage-gated potassium channels are required for proliferation and may also help to determine the final identity and morphology of the cell [19–22]. The results of experiments in lymphocytes where the inhibition of K^+^ channel activity induces a reversible cell-cycle arrest [23,24] or experiments where potassium channel blockers inhibit Schwann cell proliferation in a dose-dependent manner [22,25,26] have been replicated in many systems and by many approaches; data from those experiments have been compiled already in several reviews (e.g. [27–31]).

Direct evidence for a change in ion channel composition in G1 phase was obtained from embryonic retinal cells, which express mainly two membrane conductances, delayed rectifier (I_K_) and inward rectifier (I_Kir_) potassium currents [32]. Daughters of the same parental cell examined during and after mitosis always expressed similar I_K_ and I_Kir_ densities. However, non-sibling cells showed quantitative and qualitative differences in I_K_ and I_Kir_ densities. The heterogeneity therefore arises *after* cells re-enter G1, because the density distribution of potassium channels at cytokinesis is shown to be symmetric in both daughter cells [33].

The mechanisms controlling ion channel densities along the cell cycle appear to be manifold. For example, K^+^ channel activity in mouse oocytes is at least partly independent of the nuclear cell-cycle clock, because channel activity continues to cycle in bisected embryos in the anucleate as well as the nucleate fragments [34]. This suggests the active contribution of the cytoplasmic cell-cycle clock, which may involve changes induced by surface contractions and deformations before the cleavage of daughter cells on the channel activity [34,35]. Thus, potassium channels are proposed to be involved in the signal transduction elicited by cell-cycle checkpoints, and help to elicit cell responses in the cell-cycle machinery, integrating the nuclear clock and the cytoplasmic cell-cycle clock. Pointing towards this hypothesis, there have been reports where K^+^ channel blockers (TEA) and depolarizing agents (veratridine) inhibit cell proliferation in oligodendrocyte progenitors in cell culture and cerebellar tissue slices, inducing G1 arrest through accumulation of p27^kip1^ and p21^CIP1^, two CDK inhibitors known to regulate cell proliferation [36,37].

## 4. Importance of K^+^ channels relies on both ionic conduction and permeation-independent mechanisms

The participation of K^+^ channels in the control of cell cycle could be an early event in evolution. The pore structure and the selectivity filter have been conserved between the prokaryotic and eukaryotic K^+^ channels [38], which suggests that they evolved very early. The importance of K^+^ channels in the cell-cycle progression can also be illustrated in plant cells, for which K^+^ is a major nutrient. BY-2 tobacco cells require an increase in the K^+^ concentration in order to re-enter the cell cycle. The elevated K^+^ concentration increases the turgor pressure, which is required for cell growth. This is achieved by the activity of the inward rectifier K^+^ uptake channels [39]. By contrast, mitosis requires a transient decrease in turgor pressure owing to K^+^ efflux channels. In what could be a reminiscence of this function, the role of K^+^ channels in homoeostatic cell volume regulation is well established, and they play a role in cell volume changes along the cell cycle [40,41]. For instance, in a subset of human medulloblastomas, a voltage-gated K^+^ channel (K_V_10.2) seems to be required for the completion of mitosis, because it participates in cell volume reduction prior to cytokinesis [21].

K^+^ channels also provide the driving force required for Ca^2+^ to enter the cell by shifting the membrane potential towards negative values. Ca^2+^ is an important mediator of intracellular signals implicated in the control of proliferation among other crucial processes in cell physiology, and by keeping the membrane potential at hyperpolarized values, K^+^ channels ensure efficient Ca^2+^ entry into the cell [42–45]. Still, regardless of whether the potassium gradient is used to generate driving force for Ca^2+^ or to change the cell volume, we traditionally tend to define the potassium current as the only effector, and ignore possible additional actions of the ion channel molecule itself. If only K^+^ flow was required, essentially any potassium channel expressed at the right moment would be able to affect cell-cycle progression. Experimental observations using either siRNA knockdown or specific blockers, for example antibodies, have repeatedly shown, however, that a specific potassium channel can be important for proliferation (e.g. [46–50]). This would indicate a permeation-independent, non-canonical mechanism that could involve protein–protein interactions, dependent or independent of the conformational changes of the channel mediated by voltage. Non-canonical functions [51] have been described for at least the *Drosophila eag* channel [52], its mammalian orthologue K_V_10.1 [53], K_V_1.3 [54] and K_Ca_3.1 [55], which are still able influence cell proliferation in the absence of K^+^ permeation. Moreover, an alternatively spliced form of *Drosophila eag* that lacks the transmembrane regions, and therefore is not even a bona fide potassium channel has also been reported to influence intracellular signalling and alter cell morphology in the background of PKA/PKC activation [56].

**Figure 3.**
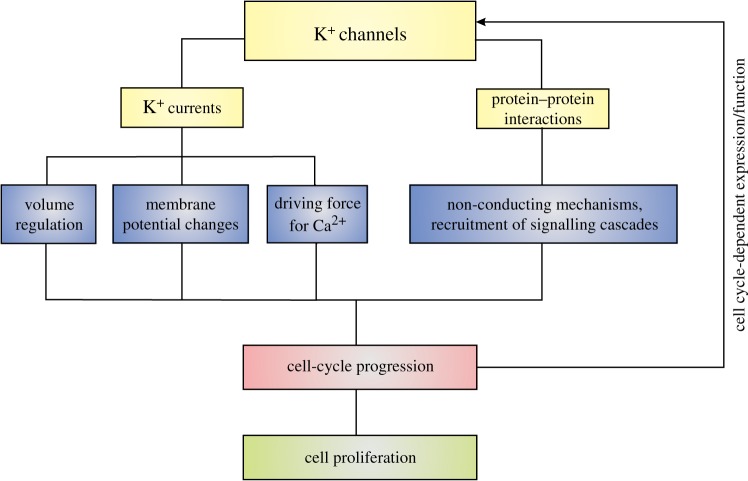
K^+^ channels influence cell-cycle progression through permeation-related and non-canonical mechanisms. The former include cell volume regulation, modulation of membrane potential and generation of driving for Ca^2+^, while the latter rely on protein–protein interactions. K^+^ channel expression or function can in turn be regulated by progression through the cell cycle.

In more general terms, the fact that more than 70 genes encode K^+^ channels suggests an exquisite distribution of functions among specific molecular entities, rather than a homogeneous function for all potassium channels. Along these lines, the variability of K^+^ channels is further increased by the formation of heteromultimers, the influence of accessory subunits and a large number of post-translational modifications, such as glycosylation [57], phosphorylation [58] and sumoylation [59]. There is substantial evidence that several K^+^ channels play a role in cell cycle and proliferation by means of both permeation-related and unrelated mechanisms (figure 3). Below, we describe some of them in more detail.

## 5. K_V_1.3

K_V_1.3 (together with K_Ca_3.1) was probably the first case showing the involvement of K^+^ channels in cell proliferation [13,60]. In a very early report on T lymphocytes, mitogenesis induced by phytohaemagglutinin caused K^+^ channels to open more rapidly and at more negative membrane potentials, suggesting that they may play a role in mitogenesis [13]. K_V_1.3 blockade was shown to suppress T-cell activation and Ca^2+^ signalling in human T cells owing to membrane depolarization, resulting in a reduced driving force for Ca^2+^ entry and impairment of activation by agents inducing mitogenesis [61,62]. K_V_1.3 can act in conjunction with K_Ca_3.1, which is a Ca^2+^-dependent K^+^ channel activated by Ca^2+^–calmodulin [63]. K_V_1.3 and K_Ca_3.1 have been found to cluster at the immunological synapse following contact with an antigen-presenting cell [60]. Together, K_V_1.3 and K_Ca_3.1 modulate calcium-dependent cellular processes in immune cells, such as T-cell activation and proliferation [43,64]. K_Ca_3.1 has also been implicated in the control of cell proliferation in rat mesenchymal stem cells, vascular smooth muscle cells (VSMCs), hepatocellular carcinoma cells as well as endometrial and prostate cancer cells [45,46,65–68], although in glioma cells K_Ca_3.1 knockdown abolished the current but did not affect proliferation [69]. As K_Ca_3.1 seems to play a crucial role in glioma cell migration [70–75], it might be difficult to dissect both properties and the results can depend very strongly on the methods used to determine proliferation.

K_V_1.3 has also been implicated in the control of the cell cycle in many other cell types, such active microglia cells [76,77], proliferating oligodendrocyte progenitors during G1/S transition [37] and macrophages [78–80]. In human endothelial cells, vascular endothelial growth factor induces a K_V_1.3-dependent hyperpolarization that results in an increased Ca^2+^ entry, which is responsible for the effects on proliferation [81,82]. It has been shown that the contractile activity of VSMCs controlling blood flow changes during the course of several vascular disorders and the cells acquire a proliferative and migratory phenotype [83]. K_V_1.3 functional expression is associated with the proliferative phenotype, because the blockade of the channel induces a significant inhibition of cell proliferation [81,84,85]. Switching from contractile to proliferative phenotype is thus associated with changes in ion channel activity. However, one study suggests K_V_1.3 increases VSMC proliferation by voltage-dependent conformational changes of the channel that activate intracellular signalling pathways, rather than by ionic conduction [54].

## 6. K_V_11.1

The voltage-sensitive human *ether à go-go*-related gene (hERG, K_V_11.1) [86] potassium channels have emerged as regulators of both proliferation and survival in cancer cells. K_V_11.1 (encoded by *KCNH2*) channel expression in normal adult human tissue is abundant in heart, brain, myometrium, pancreas and haematopoietic progenitors [87–90]. K_V_11.1 expression has been reported in many cancer types as well as cancer cell lines of different lineages, be it epithelial, leukemic, connective or neuronal [89–91] Various studies have demonstrated this expression to be largely confined to neoplastic cells both in solid and haematological malignancies, when compared with neighbouring normal tissues or normal bone marrow samples [90–94]. Studies over the past decade have also shown its expression to be preferential to the cancer stem cells especially in leukaemia when compared with normal haematopoietic stem cells [90,94]. K_V_11.1 expression has also been linked to higher grade and worse prognosis, both in the case of solid as well as haematological malignancies [89,91–94]. K_V_11.1 expression is not an epiphenomenon of cancer cells and rather plays a relevant role in their proliferative capacity, for both haematological as well as solid tumours [49,90–98]. Studies by various groups on K_V_11.1 inhibition in cell lines derived from solid tumours or leukaemias have shown a clear reduction in proliferation [49,90–99]. The reduction in cell proliferation has been explained by either increase in apoptosis or an arrest at the G0/G1 phase of cell cycle [49,90–99]. Nevertheless, the anti-tumour effects of blockers of K_V_11.1 appear to act through a reduction in cell proliferation [49,82,98,99]. Some studies have implicated the two isoforms of hERG (hERG1a and hERG1b) to play a vital role not only in cell proliferation by affecting different phases of cell cycle but also in the channel kinetics and current amplitude [100]. Both isoforms have been shown to coexist, but hERG1b expression is more prominent in the S phase of the cell cycle and hERG1a expression in the G1 phase. Modulation of these expression patterns affects the cell proliferation [95]. Co-assembly of hERG1a with hERG1b results in increased availability of channels on the plasma membrane and a larger current flow when compared with homomeric forms of the channel [100]. Further insight into the hERG isoforms and its role in cancer is needed to conclusively designate hERG as a therapeutic target.

## 7. K_V_10.1

K_V_10.1 (Eag1, encoded by *KCNH1*) is one of the best-studied ion channels in the context of cancer. Its oncogenic potential was first described in 1999 with the discovery that the inhibition of K_V_10.1 expression reduces proliferation of several somatic cancer cell lines [101]. K_V_10.1 overexpression, in turn, increases cell proliferation and can confer a transformed phenotype. In the same study, our laboratory also reported that K_V_10.1 is undetectable in healthy tissues outside the brain and favours xenograft tumour progression in immunodeficient mice *in vivo*. Along these lines, K_V_10.1 has also been detected in approximately 70% of human tumour biopsies of diverse origin [102–113]. Its widespread presence in clinical samples, together with the fact that the physiological expression of K_V_10.1 is confined to the brain (with the exception of a few restricted cell populations [111]), aroused a lot of interest in the channel owing to its potential therapeutic and diagnostic applications. It had been assumed that K_V_10.1 is present only in solid tumours but recent research has revealed its presence in leukaemias, correlating with a poor prognosis [107]. K_V_10.1 expression also correlates with poor prognosis for patients of ovarian [106], gastric [112] and colon cancer [114], and with lymph node metastasis in gastric cancer and head and neck squamous cell carcinoma, where it also correlates with the disease stage [105]. Moreover, a number of studies have supported the observation that K_V_10.1 blockage or knockdown decreases the proliferation of many cancer cell lines and *in vivo* tumour models [53,107,115,116]. An interesting exception here is glioblastoma, where the levels of K_V_10.1 are lower than that in healthy brain tissue [109], while further silencing of channel expression increases the responsiveness to interferon gamma treatment [117]. Although it is probably not the only relevant localization of K_V_10.1 [118], it is also worth mentioning here that membrane localization makes K_V_10.1 an attractive target for therapy, as it is easily accessible from the extracellular side. In order to selectively induce apoptosis in cancer cells, an anti- K_V_10.1 antibody has been coupled to TNF-related apoptosis-inducing ligand**,** and this strategy has been successfully tested *in vitro* [119].

The mechanisms of how K_V_10.1 is able to increase cell proliferation and favour tumour progression remain elusive. Ion permeation does not seem to be a necessary condition for either of the above, as non-conducting mutants retain the ability to influence proliferation and tumourigenesis [52,53]. By implication, the advantage K_V_10.1 expression confers is independent of the ‘classical’ contributions of K^+^ channels to proliferation: regulating cell volume, maintaining the driving force for Ca^2+^ and G1/S hyperpolarization. As we already indicated earlier, this is less surprising than it may appear, because if the features associated with K^+^ permeation were enough to render a transformed phenotype, many more K^+^ channels would be oncogenic. Moreover, the loss of ionic conductances can often be compensated for by other channels, which also does not fit into the picture where removing a particular conductance drastically reduces proliferation in so many cancer cell lines, as well as tumourigenesis *in vivo*. In contrast to ion permeation, voltage-dependent conformations may be crucial for K_V_10.1 to support proliferation, as the non-conducting mutants that have a preference for the open conformation fail to influence proliferation [52]. It is important to note that channel blockers could reduce proliferation not only by inhibiting permeation, but also by trapping the channel in a particular conformation. Hegle and co-workers also described an increase in p38-MAP kinase activity in non-cancer cells transfected with K_V_10.1, and abolishing the effect of K_V_10.1 on cell proliferation by p-38^MAPK^ inhibition. Interestingly, modulation of K_V_10.1 expression levels by p-38^MAPK^ pathway has been described in MG-63 cells from osteosarcoma [102], so the relation between the channel and p-38^MAPK^ signalling needs further clarification. Another non-conducting function of K_V_10.1 is an increase in hypoxia resistance by boosting HIF-1 levels and VEGF secretion, eventually leading to better tumour vascularization [53]. Nevertheless, the mechanisms described above remain insufficient to explain the benefit K_V_10.1 expression brings to the proliferation of so many different cancer cell lines.

Finally, in some models, K_V_10.1 appears to be regulated by cell cycle. Inducing the G2/M transition by progesterone in *Xenopus* oocytes heterologously expressing K_V_10.1 causes a reduction in current [17]. This reduction is dependent on the mitosis-promoting factor (MPF, a complex of cyclin B and p34^cdc2^) and obeys a voltage-dependent block by intracellular Na^+^ [16]. MPF induces an increase in selectivity during the M phase [120] that results in block by Na^+^, which leads to a rectification of the current–voltage relation. The resulting net loss of K^+^ conductance at G2/M transition may be a way to achieve membrane depolarization associated with mitosis. Cell-cycle regulation of K_V_10.1 has also been studied in MCF-7 breast cancer cells. Synchronization of these cells in G0/G1 by serum starvation leads to an increase in Eag1 mRNA expression compared with unsynchronized controls, with a further increase during the progression through G1 and a decrease in the S-phase [121]. At the functional level, this is accompanied by an increase in outward-rectifier K^+^ current that hyperpolarizes the membrane towards the S-phase [121]. Both K_V_10.1 mRNA and K_V_10.1-mediated current in MCF-7 cells can also be increased by stimulation with insulin-like growth factor 1 (IGF-1) via the PI3 K/Akt pathway, suggesting that the progression through G1 to S triggered by IGF-1 can partially be owing to its effect on K_V_10.1 [122]. Defective checkpoint control between G1 and S-phase can also result in K_V_10.1 upregulation. In SH-SY5Y neuroblastoma cells, K_V_10.1 expression is regulated by the p53/mir34/E2F1 pathway [123]. Additionally, keratinocytes immortalized with human papilloma virus oncogenes E6 and E7 targeting p53 and Retinoblastoma protein (pRb) start to transcribe K_V_10.1 mRNA [124]. One can thus expect that p53 or pRb/E2F pathway inhibition or malfunctions, which are very common in cancer, can give rise to higher K_V_10.1 expression levels. However, further research is needed to establish that K_V_10.1 expression is cell-cycle dependent and to elucidate the effect(s) of the channel on cell-cycle progression.

## 8. Conclusion

Progression through the cell cycle is guarded by several checkpoint control pathways that have the ability to delay or stop further events, such as DNA synthesis or assembly of the mitotic spindle, before commitment into cell division. In accordance with the experimental data compiled in this review, there can be little doubt that K^+^ channels play an active role in cell-cycle progression. On the other hand, their expression or function can be regulated by the cell cycle. Therefore, K^+^ channels could also be viewed as effectors of the checkpoint machinery. As molecular machines that enable the passage of K^+^ ions through the membrane, they can regulate cell volume, provide driving force for Ca^2+^ entry, hyperpolarize the cell at the G1/S transition and depolarize it towards mitosis. Additionally, non-canonical, permeation-independent mechanisms may be involved, where K^+^ channels recruit or modulate signalling cascades via protein–protein interactions. It is tempting to assume that signalling cascades activated by such interactions could link the nuclear clock control with its cytoplasmic counterpart.

Unfortunately, to date we have only a rough estimate of how membrane potential changes along the cell cycle. Moreover, very little is known about the non-conducting functions of K^+^ channels. Which signalling cascades can they modify? How do they interact with other proteins? There are also more general questions that remain unanswered. How exactly does membrane potential affect the cell-cycle machinery? Further research on K^+^ channels in cell cycle and proliferation will give us better understanding of these fundamental processes and may have therapeutic implications.

## References

[1] Hartwell LH Culotti J Pringle JR Reid BJ 1974 Genetic control of the cell division cycle in yeast. Science 183, 46–51. (10.1126/science.183.4120.46) 4587263

[2] Hartwell LH Weinert TA 1989 Checkpoints: controls that ensure the order of cell cycle events. Science 246, 629–634. (10.1126/science.2683079) 2683079

[3] Murray AW 1992 Creative blocks: cell-cycle checkpoints and feedback controls. Nature 359, 599–604. (10.1038/359599a0) 1406993

[4] Foster DA Yellen P Xu L Saqcena M 2010 Regulation of G1 cell cycle progression: distinguishing the restriction point from a nutrient-sensing cell growth checkpoint(s). Genes Cancer 1, 1124–1131. (10.1177/1947601910392989) 21779436 PMC3092273

[5] Rieder CL 2011 Mitosis in vertebrates: the G2/M and M/A transitions and their associated checkpoints. Chromosome Res. 19, 291–306. (10.1007/s10577-010-9178-z) 21194009

[6] Blackiston DJ McLaughlin KA Levin M 2009 Bioelectric controls of cell proliferation: ion channels, membrane voltage and the cell cycle. Cell cycle 8, 3519–3528. (10.4161/cc.8.21.9888) PMC2862582 19823012

[7] Cone CD Jr 1971 Unified theory on the basic mechanism of normal mitotic control and oncogenesis. J. Theor. Biol. 30, 151–181. (10.1016/0022-5193(71)90042-7) 5555269

[8] Stillwell EF Cone CM Cone CD Jr 1973 Stimulation of DNA synthesis in CNS neurones by sustained depolarisation. Nat. New Biol. 246, 110–111. (10.1038/newbio246110a0) 4518935

[9] Cone CD Jr Cone CM 1976 Induction of mitosis in mature neurons in central nervous system by sustained depolarization. Science 192, 155–158. (10.1126/science.56781) 56781

[10] Boonstra J Mummery CL Tertoolen LG Van Der Saag PT De Laat SW 1981 Cation transport and growth regulation in neuroblastoma cells: modulations of K^+^ transport and electrical membrane properties during the cell cycle. J. Cell. Physiol. 107, 75–83. (10.1002/jcp.1041070110) 7217225

[11] Sachs HG Stambrook PJ Ebert JD 1974 Changes in membrane potential during the cell cycle. Exp. Cell Res. 83, 362–366. (10.1016/0014-4827(74)90350-4) 4856272

[12] Wonderlin WF Woodfork KA Strobl JS 1995 Changes in membrane potential during the progression of MCF-7 human mammary tumor cells through the cell cycle. J. Cell. Physiol. 165, 177–185. (10.1002/jcp.1041650121) 7559799

[13] DeCoursey TE Chandy KG Gupta S Cahalan MD 1984 Voltage-gated K^+^ channels in human T lymphocytes: a role in mitogenesis? Nature 307, 465–468. (10.1038/307465a0) 6320007

[14] Takahashi A Yamaguchi H Miyamoto H 1993 Change in K^+^ current of HeLa cells with progression of the cell cycle studied by patch-clamp technique. Am. J. Physiol. 265 C328–C336. 8368262 10.1152/ajpcell.1993.265.2.C328

[15] Arcangeli A Bianchi L Becchetti A Faravelli L Coronnello M Mini E Olivotto M Wanke E 1995 A novel inward-rectifying K^+^ current with a cell-cycle dependence governs the resting potential of mammalian neuroblastoma cells. J. Physiol. Lond. 489, 455–471. 8847640 10.1113/jphysiol.1995.sp021065 PMC1156772

[16] Pardo LA Bruggemann A Camacho J Stuhmer W 1998 Cell cycle-related changes in the conducting properties of r-eag K^+^ channels. J. Cell Biol. 143, 767–775. (10.1083/jcb.143.3.767) 9813096 PMC2148139

[17] Bruggemann A Stuhmer W Pardo LA 1997 Mitosis-promoting factor-mediated suppression of a cloned delayed rectifier potassium channel expressed in *Xenopus* oocytes. Proc. Natl Acad. Sci. USA 94, 537–542. (10.1073/pnas.94.2.537) 9012819 PMC19548

[18] Day ML Pickering SJ Johnson MH Cook DI 1993 Cell-cycle control of a large-conductance K^+^ channel in mouse early embryos. Nature 365, 560–562. (10.1038/365560a0) 8413614

[19] Becchetti A 2011 Ion channels and transporters in cancer. 1. Ion channels and cell proliferation in cancer. Am. J. Physiol. Cell Physiol. 301, C255–C265. (10.1152/ajpcell.00047.2011) 21430288

[20] Wonderlin WF Strobl JS 1996 Potassium channels, proliferation and G1 progression. J. Membr. Biol. 154, 91–107. (10.1007/s002329900135) 8929284

[21] Huang X 2012 Voltage-gated potassium channel EAG2 controls mitotic entry and tumor growth in medulloblastoma via regulating cell volume dynamics. Genes Dev. 26, 1780–1796. (10.1101/gad.193789.112) 22855790 PMC3426758

[22] Chiu SY Wilson GF 1989 The role of potassium channels in Schwann cell proliferation in Wallerian degeneration of explant rabbit sciatic nerves. J. Physiol. 408, 199–222. 2476555 10.1113/jphysiol.1989.sp017455 PMC1190399

[23] Chandy KG DeCoursey TE Cahalan MD McLaughlin C Gupta S 1984 Voltage-gated potassium channels are required for human T lymphocyte activation. J. Exp. Med. 160, 369–385. (10.1084/jem.160.2.369) 6088661 PMC2187449

[24] Amigorena S Choquet D Teillaud JL Korn H Fridman WH 1990 Ion channel blockers inhibit B cell activation at a precise stage of the G1 phase of the cell cycle. Possible involvement of K^+^ channels. J. Immunol. 144, 2038–2045. 2313087

[25] Wilson GF Chiu SY 1993 Mitogenic factors regulate ion channels in Schwann cells cultured from newborn rat sciatic nerve. J. Physiol. 470, 501–520. 7508507 10.1113/jphysiol.1993.sp019872 PMC1143931

[26] Fieber LA Gonzalez DM Wallace MR Muir D 2003 Delayed rectifier K currents in NF1 Schwann cells. Pharmacological block inhibits proliferation. Neurobiol. Dis. 13, 136–146. (10.1016/S0969-9961(03)00031-7) 12828937

[27] Prevarskaya N Skryma R Bidaux G Flourakis M Shuba Y 2007 Ion channels in death and differentiation of prostate cancer cells. Cell Death Differ. 14, 1295–1304. (10.1038/sj.cdd.4402162) 17479110

[28] Villalonga N Ferreres JC Argiles JM Condom E Felipe A 2007 Potassium channels are a new target field in anticancer drug design. Recent Patents Anticancer Drug Disc. 2, 212–223. (10.2174/157489207782497181) 18221064

[29] Arcangeli A Crociani O Lastraioli E Masi A Pillozzi S Becchetti A 2009 Targeting ion channels in cancer: a novel frontier in antineoplastic therapy. Curr. Med. Chem. 16, 66–93. (10.2174/092986709787002835) 19149563

[30] Arcangeli A Pillozzi S Becchetti A 2012 Targeting ion channels in leukemias: a new challenge for treatment. Curr. Med. Chem. 19, 683–696. (10.2174/092986712798992093) 22204341

[31] Huber SM 2013 Oncochannels. Cell Calcium 53, 241–255. (10.1016/j.ceca.2013.01.001) 23357407

[32] Lenzi D Radke K Wilson M 1991 Clonal cells from embryonic retinal cell lines express qualitative electrophysiological differences. J. Neurobiol. 22, 823–836. (10.1002/neu.480220804) 1723422

[33] Lenzi D Radke K Wilson M 1993 Symmetrical segregation of potassium channels at cytokinesis. J. Neurobiol. 24, 675–686. (10.1002/neu.480240511) 7686965

[34] Day ML Johnson MH Cook DI 1998 A cytoplasmic cell cycle controls the activity of a K^+^ channel in pre-implantation mouse embryos. EMBO J. 17, 1952–1960. (10.1093/emboj/17.7.1952) 9524118 PMC1170541

[35] Ciemerych MA 1995 Chromatin condensation activity and cortical activity during the first three cell cycles of a mouse embryo. Mol. Reprod. Dev. 41, 416–424. (10.1002/mrd.1080410404) 7576609

[36] Ghiani CA Yuan X Eisen AM Knutson PL DePinho RA McBain CJ Gallo V 1999 Voltage-activated K^+^ channels and membrane depolarization regulate accumulation of the cyclin-dependent kinase inhibitors p27(Kip1) and p21(CIP1) in glial progenitor cells. J. Neurosci. 19, 5380–5392. 10377348 10.1523/JNEUROSCI.19-13-05380.1999 PMC6782320

[37] Chittajallu R Chen Y Wang H Yuan X Ghiani CA Heckman T McBain CJ Gallo V 2002 Regulation of Kv1 subunit expression in oligodendrocyte progenitor cells and their role in G(1)/S phase progression of the cell cycle. Proc. Natl Acad. Sci. USA 99, 2350–2355. (10.1073/pnas.042698399) 11854528 PMC122368

[38] Doyle DA Morais Cabral J Pfuetzner RA Kuo A Gulbis JM Cohen SL Chait BT MacKinnon R 1998 The structure of the potassium channel: molecular basis of K^+^ conduction and selectivity. Science 280, 69–77. (10.1126/science.280.5360.69) 9525859

[39] Sano T Becker D Ivashikina N Wegner LH Zimmermann U Roelfsema MR Nagata T Hedrich R 2007 Plant cells must pass a K^+^ threshold to re-enter the cell cycle. Plant J. 50, 401–413. (10.1111/j.1365-313X.2007.03071.x) 17425714

[40] Rouzaire-Dubois B Dubois JM 1991 A quantitative analysis of the role of K^+^ channels in mitogenesis of neuroblastoma cells. Cell. Signal. 3, 333–339. (10.1016/0898-6568(91)90062-Y) 1931483

[41] Dubois JM Rouzaire-Dubois B 2004 The influence of cell volume changes on tumour cell proliferation. Eur. Biophys. J. 33, 227–232. (10.1007/s00249-003-0364-1) 14598000

[42] Lee YS Sayeed MM Wurster RD 1993 Inhibition of cell growth by K^+^ channel modulators is due to interference with agonist-induced Ca^2+^ release. Cell. Signal. 5, 803–809. (10.1016/0898-6568(93)90041-J) 8130083

[43] Lin CS 1993 Voltage-gated potassium channels regulate calcium-dependent pathways involved in human T lymphocyte activation. J. Exp. Med. 177, 637–645. (10.1084/jem.177.3.637) 7679705 PMC2190940

[44] Lepple-Wienhues A Berweck S Bohmig M Leo CP Meyling B Garbe C Wiederholt M 1996 K^+^ channels and the intracellular calcium signal in human melanoma cell proliferation. J. Membr. Biol. 151, 149–157. (10.1007/s002329900066) 8661503

[45] Lallet-Daher H 2009 Intermediate-conductance Ca^2+^-activated K^+^ channels (IKCa1) regulate human prostate cancer cell proliferation through a close control of calcium entry. Oncogene 28, 1792–1806. (10.1038/onc.2009.25) 19270724

[46] Wang ZH Shen B Yao HL Jia YC Ren J Feng YJ Wang YZ 2007 Blockage of intermediate-conductance-Ca^2+^-activated K^+^ channels inhibits progression of human endometrial cancer. Oncogene 26, 5107–5114. (10.1038/sj.onc.1210308) 17310992

[47] Jang SH 2009 Silencing of Kv4.1 potassium channels inhibits cell proliferation of tumorigenic human mammary epithelial cells. Biochem. Biophys. Res. Commun. 384, 180–186. (10.1016/j.bbrc.2009.04.108) 19401188

[48] Miguel-Velado E Perez-Carretero FD Colinas O Cidad P Heras M Lopez-Lopez JR Perez-Garcia MT 2010 Cell cycle-dependent expression of Kv3.4 channels modulates proliferation of human uterine artery smooth muscle cells. Cardiovasc. Res. 86, 383–391. (10.1093/cvr/cvq011) 20093253

[49] Glassmeier G Hempel K Wulfsen I Bauer CK Schumacher U Schwarz JR 2012 Inhibition of HERG1 K^+^ channel protein expression decreases cell proliferation of human small cell lung cancer cells. Pflugers Arch. 463, 365–376. (10.1007/s00424-011-1045-z) 22075718 PMC3261411

[50] Yasuda T Cuny H Adams DJ 2013 Kv3.1 channels stimulate adult neural precursor cell proliferation and neuronal differentiation. J. Physiol. 591, 2579–2591. (10.1113/jphysiol.2012.249151) 23478135 PMC3678044

[51] Kaczmarek LK 2006 Non-conducting functions of voltage-gated ion channels. Nat. Rev. Neurosci. 7, 761–771. (10.1038/nrn1988) 16988652

[52] Hegle AP Marble DD Wilson GF 2006 A voltage-driven switch for ion-independent signaling by ether-a-go-go K^+^ channels. Proc. Natl Acad. Sci. USA 103, 2886–2891. (10.1073/pnas.0505909103) 16477030 PMC1413768

[53] Downie BR Sanchez A Knotgen H Contreras-Jurado C Gymnopoulos M Weber C Stuhmer W Pardo LA 2008 Eag1 Expression Interferes with hypoxia homeostasis and induces angiogenesis in tumors. J. Biol. Chem. 283, 36 234–36 240. (10.1074/Jbc.M801830200) PMC2606018 18927085

[54] Cidad P Jimenez-Perez L Garcia-Arribas D Miguel-Velado E Tajada S Ruiz-McDavitt C Lopez-Lopez JR Perez-Garcia MT 2012 Kv1.3 channels can modulate cell proliferation during phenotypic switch by an ion-flux independent mechanism. Arterioscler. Thromb. Vasc. Biol. 32, 1299–1307. (10.1161/atvbaha.111.242727) 22383699

[55] Millership JE Devor DC Hamilton KL Balut CM Bruce JI Fearon IM 2011 Calcium-activated K^+^ channels increase cell proliferation independent of K^+^ conductance. Am. J. Physiol. Cell Physiol. 300, C792–C802. (10.1152/ajpcell.00274.2010) 21123738 PMC3074627

[56] Itoh T Hasegawa J Tsujita K Kanaho Y Takenawa T 2009 The tyrosine kinase Fer is a downstream target of the PLD-PA pathway that regulates cell migration. Sci. Signal. 2, ra52 (10.1126/scisignal.2000393) 19738202

[57] Norring SA Ednie AR Schwetz TA Du D Yang H Bennett ES 2013 Channel sialic acids limit hERG channel activity during the ventricular action potential. FASEB J. 27, 622–631. (10.1096/fj.12-214387) 23139156

[58] Lang F Shumilina E 2013 Regulation of ion channels by the serum- and glucocorticoid-inducible kinase SGK1. FASEB J. 27, 3–12. (10.1096/fj.12-218230) 23012321

[59] Benson MD Li QJ Kieckhafer K Dudek D Whorton MR Sunahara RK Iniguez-Lluhi JA Martens JR 2007 SUMO modification regulates inactivation of the voltage-gated potassium channel Kv1.5. Proc. Natl Acad. Sci. USA 104, 1805–1810. (10.1073/pnas.0606702104) 17261810 PMC1794304

[60] Cahalan MD Chandy KG 2009 The functional network of ion channels in T lymphocytes. Immunol. Rev. 231, 59–87. (10.1111/j.1600-065X.2009.00816.x) 19754890 PMC3133616

[61] Lam J Wulff H 2011 The lymphocyte potassium channels Kv1.3 and KCa3.1 as targets for immunosuppression. Drug Dev. Res. 72, 573–584. (10.1002/ddr.20467) 22241939 PMC3253536

[62] Leonard RJ Garcia ML Slaughter RS Reuben JP 1992 Selective blockers of voltage-gated K^+^ channels depolarize human T lymphocytes: mechanism of the antiproliferative effect of charybdotoxin. Proc. Natl Acad. Sci. USA 89, 10 094–10 098. (10.1073/pnas.89.21.10094) PMC50284 1279670

[63] Joiner WJ Khanna R Schlichter LC Kaczmarek LK 2001 Calmodulin regulates assembly and trafficking of SK4/IK1 Ca^2+^-activated K^+^ channels. J. Biol. Chem. 276, 37 980–37 985. 10.1074/jbc.M104965200 11495911

[64] Hu J 2007 Calcium-activated potassium channels mediated blood-brain tumor barrier opening in a rat metastatic brain tumor model. Mol. Cancer 6, 22 (10.1186/1476-4598-6-22) 17359538 PMC1831484

[65] Bi D 2013 The intermediate-conductance calcium-activated potassium channel KCa3.1 regulates vascular smooth muscle cell proliferation via controlling calcium-dependent signaling. J. Biol. Chem. 288, 15 843–15 853. (10.1074/jbc.M112.427187) PMC3668741 23609438

[66] Su XL Wang Y Zhang W Zhao LM Li GR Deng XL 2011 Insulin-mediated upregulation of K(Ca)3.1 channels promotes cell migration and proliferation in rat vascular smooth muscle. J. Mol. Cell. Cardiol. 51, 51–57. (10.1016/j.yjmcc.2011.03.014) 21463632

[67] Wang SP Wang JA Luo RH Cui WY Wang H 2008 Potassium channel currents in rat mesenchymal stem cells and their possible roles in cell proliferation. Clin. Exp. Pharmacol. Physiol. 35, 1077–1084. (10.1111/j.1440-1681.2008.04964.x) 18505444

[68] Yang XW Liu JW Zhang RC Yin Q Shen WZ Yi JL 2013 Inhibitory effects of blockage of intermediate conductance Ca^2+^-activated K^+^ channels on proliferation of hepatocellular carcinoma cells. J. Huazhong Univ. Sci. Technol. Med. Sci. 33, 86–89. (10.1007/s11596-013-1076-0) 23392713

[69] Abdullaev IF Rudkouskaya A Mongin AA Kuo YH 2010 Calcium-activated potassium channels BK and IK1 are functionally expressed in human gliomas but do not regulate cell proliferation. PLoS ONE 5, e12304 (10.1371/journal.pone.0012304) 20808839 PMC2924897

[70] Schwab A Nechyporuk-Zloy V Gassner B Schulz C Kessler W Mally S Romer M Stock C 2012 Dynamic redistribution of calcium sensitive potassium channels (hK(Ca)3.1) in migrating cells. J. Cell. Physiol. 227, 686–696. (10.1002/jcp.22776) 21465474

[71] Schwab A Reinhardt J Schneider SW Gassner B Schuricht B 1999 K^+^ channel-dependent migration of fibroblasts and human melanoma cells. Cell. Physiol. Biochem. 9, 126–132. (10.1159/000016309) 10494026

[72] Schwab A Wojnowski L Gabriel K Oberleithner H 1994 Oscillating activity of a Ca^2+^-sensitive K^+^ channel. A prerequisite for migration of transformed Madin-Darby canine kidney focus cells. J. Clin. Investig. 93, 1631–1636. (10.1172/JCI117144) 8163666 PMC294199

[73] Schwab A 2006 Subcellular distribution of calcium-sensitive potassium channels (IK1) in migrating cells. J. Cell. Physiol. 206, 86–94. (10.1002/jcp.20434) 15965951

[74] Catacuzzeno L Aiello F Fioretti B Sforna L Castigli E Ruggieri P Tata AM Calogero A Franciolini F 2011 Serum-activated K and Cl currents underlay U87-MG glioblastoma cell migration. J. Cell. Physiol. 226, 1926–1933. (10.1002/jcp.22523) 21506123

[75] Ruggieri P 2012 The inhibition of KCa3.1 channels activity reduces cell motility in glioblastoma derived cancer stem cells. PLoS ONE 7, e47825 (10.1371/journal.pone.0047825) 23110108 PMC3478269

[76] Kotecha SA Schlichter LC 1999 A Kv1.5 to Kv1.3 switch in endogenous hippocampal microglia and a role in proliferation. J. Neurosci. 19, 10 680–10 693. 10594052 10.1523/JNEUROSCI.19-24-10680.1999 PMC6784954

[77] Fordyce CB Jagasia R Zhu X Schlichter LC 2005 Microglia Kv1.3 channels contribute to their ability to kill neurons. J. Neurosci. 25, 7139–7149. (10.1523/jneurosci.1251-05.2005) 16079396 PMC6725234

[78] Villalonga N David M Bielanska J Gonzalez T Parra D Soler C Comes N Valenzuela C Felipe A 2010 Immunomodulatory effects of diclofenac in leukocytes through the targeting of Kv1.3 voltage-dependent potassium channels. Biochem. Pharmacol. 80, 858–866. (10.1016/j.bcp.2010.05.012) 20488163

[79] Villalonga N Escalada A Vicente R Sanchez-Tillo E Celada A Solsona C Felipe A 2007 Kv1.3/Kv1.5 heteromeric channels compromise pharmacological responses in macrophages. Biochem. Biophys. Res. Commun. 352, 913–918. (10.1016/j.bbrc.2006.11.120) 17157812

[80] Vicente R 2003 Differential voltage-dependent K^+^ channel responses during proliferation and activation in macrophages. J. Biol. Chem. 278, 46 307–46 320. (10.1074/jbc.M304388200) 12923194

[81] Erdogan A 2005 Margatoxin inhibits VEGF-induced hyperpolarization, proliferation and nitric oxide production of human endothelial cells. J. Vasc. Res. 42, 368–376. (10.1159/000087159) 16043967

[82] Li H Liu L Guo L Zhang J Du W Li X Liu W Chen X Huang S 2008 HERG K^+^ channel expression in CD34^+^/CD38^−^/CD123(high) cells and primary leukemia cells and analysis of its regulation in leukemia cells. Int. J. Hematol. 87, 387–392. (10.1007/s12185-008-0056-9) 18415658

[83] Moudgil R Michelakis ED Archer SL 2006 The role of K^+^ channels in determining pulmonary vascular tone, oxygen sensing, cell proliferation, and apoptosis: implications in hypoxic pulmonary vasoconstriction and pulmonary arterial hypertension. Microcirculation 13, 615–632. (10.1080/10739680600930222) 17085423

[84] Tian Y Yue X Luo D Wazir R Wang J Wu T Chen L Liao B Wang K 2013 Increased proliferation of human bladder smooth muscle cells is mediated by physiological cyclic stretch via the PI3KSGK1Kv1.3 pathway. Mol. Med. Rep. 8, 294–298. (10.3892/mmr.2013.1473) 23669863

[85] Cheong A 2011 Potent suppression of vascular smooth muscle cell migration and human neointimal hyperplasia by K_V_1.3 channel blockers. Cardiovasc. Res. 89, 282–289. (10.1093/cvr/cvq305) 20884640 PMC3020133

[86] Warmke JW Ganetzki B 1994 A family of potassium channel genes related to eag in *Drosophila* and mammals. Proc. Natl Acad. Sci. USA 91, 3438–3442. (10.1073/pnas.91.8.3438) 8159766 PMC43592

[87] Pond AL Scheve BK Benedict AT Petrecca K Van Wagoner DR Shrier A Nerbonne JM 2000 Expression of distinct ERG proteins in rat, mouse, and human heart - Relation to functional I-Kr channels. J. Biol. Chem. 275, 5997–6006. (10.1074/jbc.275.8.5997) 10681594

[88] Rosati B Marchetti P Crociani O Lecchi M Lupi R Arcangeli A Olivotto M Wanke E 2000 Glucose- and arginine-induced insulin secretion by human pancreatic beta-cells: the role of HERG K^+^ channels in firing and release. FASEB J. 14, 2601–2610. (10.1096/fj.00-0077com) 11099479

[89] Cherubini A 2000 HERG potassium channels are more frequently expressed in human endometrial cancer as compared to non-cancerous endometrium. Br. J. Cancer 83, 1722–1729. (10.1054/bjoc.2000.1497) 11104572 PMC2363441

[90] Pillozzi S 2002 HERG potassium channels are constitutively expressed in primary human acute myeloid leukemias and regulate cell proliferation of normal and leukemic hemopoietic progenitors. Leukemia 16, 1791–1798. (10.1038/sj.leu.2402572) 12200695

[91] Lastraioli E 2004 herg1 gene and HERG1 protein are overexpressed in colorectal cancers and regulate cell invasion of tumor cells. Cancer Res. 64, 606–611. (10.1158/0008-5472.CAN-03-2360) 14744775

[92] Ding XW Yang WB Gao S Wang W Li Z Hu WM Li JJ Luo HS 2010 Prognostic significance of hERG1 expression in gastric cancer. Dig. Dis. Sci. 55, 1004–1010. (10.1007/s10620-009-0834-0) 19495974

[93] Shao XD Wu KC Guo XZ Xie MJ Zhang J Fan DM 2008 Expression and significance of HERG protein in gastric cancer. Cancer Biol. Ther. 7, 45–50. (10.4161/cbt.7.1.5126) 17938585

[94] Smith GAM Tsui HW Newell EW Jiang XP Zhu XP Tsui FWL Schlichter LC 2002 Functional up-regulation of HERG K^+^ channels in neoplastic hematopoietic cells. J. Biol. Chem. 277, 18 528–18 534. (10.1074/jbc.M200592200) 11893742

[95] Crociani O Guasti L Balzi M Becchetti A Wanke E Olivotto M Wymore RS Arcangeli A 2003 Cell cycle-dependent expression of HERG1 and HERG1B isoforms in tumor cells. J. Biol. Chem. 278, 2947–2955. (10.1074/jbc.M210789200) 12431979

[96] Wang H Zhang Y Cao L Han H Wang J Yang B Nattel S Wang Z 2002 HERG K^+^ channel, a regulator of tumor cell apoptosis and proliferation. Cancer Res. 62, 4843–4848. 12208728

[97] Zhao J Wei XL Jia YS Zheng JQ 2008 Silencing of *herg* gene by shRNA inhibits SH-SY5Y cell growth *in vitro* and *in vivo*. Eur. J. Pharmacol. 579, 50–57. (10.1016/j.ejphar.2007.10.008) 17976575

[98] Afrasiabi E Hietamaki M Viitanen T Sukumaran P Bergelin N Tornquist K 2010 Expression and significance of HERG (KCNH2) potassium channels in the regulation of MDA-MB-435S melanoma cell proliferation and migration. Cell. Signal. 22, 57–64. (10.1016/j.cellsig.2009.09.010) 19765650

[99] Cui G Shu W Wu Q Chen Y 2009 Effect of Gambogic acid on the regulation of hERG channel in K562 cells in vitro. J. Huazhong Univ. Sci. Technol. Med. Sci. 29, 540–545. (10.1007/s11596-009-0503-8) 19821083

[100] Guasti L 2008 Identification of a posttranslational mechanism for the regulation of hERG1 K^+^ channel expression and hERG1 current density in tumor cells. Mol. Cell. Biol. 28, 5043–5060. (10.1128/MCB.00304-08) 18559421 PMC2519704

[101] Pardo LA del Camino D Sanchez A Alves F Bruggemann A Beckh S Stuhmer W 1999 Oncogenic potential of EAG K+ channels. EMBO J. 18, 5540–5547. (10.1093/emboj/18.20.5540) 10523298 PMC1171622

[102] Wu X Zhong D Lin B Zhai W Ding Z Wu J 2013 p38 MAPK regulates the expression of ether a go–go potassium channel in human osteosarcoma cells. Radiol. Oncol. 47, 42–49. (10.2478/v10019-012-0043-x) 23450231 PMC3573833

[103] del Pliego MG 2013 Expression of Eag1 K^+^ channel and ErbBs in human pituitary adenomas: cytoskeleton arrangement patterns in cultured cells. Int. J. Clin. Exp. Pathol. 6, 458–468. 23413122 PMC3563198

[104] Bai Y Liao H Liu T Zeng X Xiao F Luo L Guo H Guo L 2013 MiR-296–3p regulates cell growth and multi-drug resistance of human glioblastoma by targeting ether-a-go-go (EAG1). Eur. J. Cancer 49, 710–724. (10.1016/j.ejca.2012.08.020) 22999387

[105] Menendez ST 2012 Frequent aberrant expression of the human ether a go-go (hEAG1) potassium channel in head and neck cancer: pathobiological mechanisms and clinical implications. J. Mol. Med. 90, 1173–1184. (10.1007/s00109-012-0893-0) 22466864

[106] Asher V Khan R Warren A Shaw R Schalkwyk GV Bali A Sowter HM 2010 The Eag potassium channel as a new prognostic marker in ovarian cancer. Diagn. Pathol. 5, 78 (10.1186/1746-1596-5-78) 21138547 PMC3016344

[107] Agarwal JR Griesinger F Stuhmer W Pardo LA 2010 The potassium channel Ether a go-go is a novel prognostic factor with functional relevance in acute myeloid leukemia. Mol Cancer 9, 18 (10.1186/1476-4598-9-18) 20105281 PMC2835655

[108] Asher V Sowter H Shaw R Bali A Khan R 2010 Eag and HERG potassium channels as novel therapeutic targets in cancer. World J. Surg. Oncol. 8, 113 (10.1186/1477-7819-8-113) 21190577 PMC3022597

[109] Patt S Preussat K Beetz C Kraft R Schrey M Kalff R Schonherr K Heinemann SH 2004 Expression of ether a go-go potassium channels in human gliomas. Neurosci. Lett. 368, 249–253. (10.1016/j.neulet.2004.07.001) 15364405

[110] de Queiroz FM Suarez-Kurtz G Stuhmer W Pardo LA 2006 Ether a go-go potassium channel expression in soft tissue sarcoma patients. Mol. Cancer 5, 42 (10.1186/1476-4598-5-42) 17022811 PMC1618397

[111] Hemmerlein B 2006 Overexpression of EagI potassium channels in clinical tumours. Mol Cancer 5, 42 (10.1186/1476-4598-5-41) 17022810 PMC1621079

[112] Ding XW Luo HS Jin X Yan JJ Ai YW 2007 Aberrant expression of Eag1 potassium channels in gastric cancer patients and cell lines. Med. Oncol. 24, 345–350. (10.1007/s12032-007-0015-y) 17873312

[113] Ding XW Yan JJ An P Lu P Luo HS 2007 Aberrant expression of ether a go-go potassium channel in colorectal cancer patients and cell lines. World J. Gastroenterol. 13, 1257–1261. 17451210 10.3748/wjg.v13.i8.1257 PMC4147004

[114] Ousingsawat J Spitzner M Puntheeranurak S Terracciano L Tornillo L Bubendorf L Kunzelmann K Schreiber R 2007 Expression of voltage-gated potassium channels in human and mouse colonic carcinoma. Clin. Cancer Res. 13, 824–831. (10.1158/1078-0432.ccr-06-1940) 17289873

[115] Gomez-Varela D 2007 Monoclonal antibody blockade of the human Eag1 potassium channel function exerts antitumor activity. Cancer Res. 67, 7343–7349. (10.1158/0008-5472.Can-07-0107) 17671204

[116] Weber C de Queiroz FM Downie BR Suckow A Stuhmer W Pardo LA 2006 Silencing the activity and proliferative properties of the human EagI potassium channel by RNA interference. J. Biol. Chem. 281, 13 030–13 037. (10.1074/Jbc.M600883200) 16537547

[117] Cunha LC Del Bel E Pardo L Stuhmer W Titze DEAR 2013 RNA interference with EAG1 enhances interferon gamma injury to glioma cells *in vitro*. Anticancer Res. 33, 865–870. 23482755

[118] Chen Y Sanchez A Rubio ME Kohl T Pardo LA Stuhmer W 2011 Functional K(v)10.1 channels localize to the inner nuclear membrane. PLoS ONE 6, e19257 (10.1371/journal.pone.0019257) 21559285 PMC3086910

[119] Hartung F Stuhmer W Pardo LA 2011 Tumor cell-selective apoptosis induction through targeting of K(V)10.1 via bifunctional TRAIL antibody. Mol. Cancer 10, 109 (10.1186/1476-4598-10-109) 21899742 PMC3179451

[120] Camacho J Sanchez A Stuhmer W Pardo LA 2000 Cytoskeletal interactions determine the electrophysiological properties of human EAG potassium channels. Pflugers Arch. Eur. J. Phys. 441, 167–174. (10.1007/s004240000420) 11211100

[121] Ouadid-Ahidouch H Le Bourhis X Roudbaraki M Toillon RA Delcourt P Prevarskaya N 2001 Changes in the K^+^ current-density of MCF-7 cells during progression through the cell cycle: possible involvement of a h-ether.a-gogo K^+^ channel. Receptors Channels 7, 345–356. 11697078

[122] Borowiec AS Hague F Harir N Guenin S Guerineau F Gouilleux F Roudbaraki M Lassoued K Ouadid-Ahidouch H 2007 IGF-1 activates hEAG K^+^ channels through an Akt-dependent signaling pathway in breast cancer cells: role in cell proliferation. J. Cell. Physiol. 212, 690–701. (10.1002/jcp.21065) 17520698

[123] Lin H Li Z Chen C Luo X Xiao J Dong D Lu Y Yang B Wang Z 2011 Transcriptional and post-transcriptional mechanisms for oncogenic overexpression of ether a go-go K^+^ channel. PLoS ONE 6, e20362 (10.1371/journal.pone.0020362) 21655246 PMC3105031

[124] Diaz L 2009 Estrogens and human papilloma virus oncogenes regulate human ether-a-go-go-1 potassium channel expression. Cancer Res. 69, 3300–3307. (10.1158/0008-5472.can-08-2036) 19351862

